# Varicellovirus UL49.5 Proteins Differentially Affect the Function of the Transporter Associated with Antigen Processing, TAP

**DOI:** 10.1371/journal.ppat.1000080

**Published:** 2008-05-30

**Authors:** Danijela Koppers-Lalic, Marieke C. Verweij, Andrea D. Lipińska, Ying Wang, Edwin Quinten, Eric A. Reits, Joachim Koch, Sandra Loch, Marisa Marcondes Rezende, Franz Daus, Krystyna Bieńkowska-Szewczyk, Nikolaus Osterrieder, Thomas C. Mettenleiter, Mirjam H. M. Heemskerk, Robert Tampé, Jacques J. Neefjes, Shafiqul I. Chowdhury, Maaike E. Ressing, Frans A. M. Rijsewijk, Emmanuel J. H. J. Wiertz

**Affiliations:** 1 Center of Infectious Diseases and Department of Medical Microbiology, Leiden University Medical Center, Leiden, The Netherlands; 2 Department of Molecular Virology, University of Gdańsk, Gdańsk, Poland; 3 Department of Diagnostic Medicine/Pathobiology, College of Veterinary Medicine, Kansas State University, Manhattan, Kansas, United States of America; 4 Department of Cell Biology and Histology, Academic Medical Centre, Amsterdam, The Netherlands; 5 Institute of Biochemistry, Biocenter, Goethe-University Frankfurt, Frankfurt/Main, Germany; 6 Virus Discovery Unit, ASG-Lelystad, Lelystad, The Netherlands; 7 Department of Microbiology and Immunology, College of Veterinary Medicine, Cornell University, Ithaca, New York, United States of America; 8 Institute for Virology, Berlin, Germany; 9 Institute of Molecular Biology, Friedrich-Loeffler-Institut, Greifswald-Insel Riems, Germany; 10 Department of Hematology, Leiden University Medical Center, Leiden, The Netherlands; 11 Department of Tumor Biology, The Netherlands Cancer Institute, Amsterdam, The Netherlands; University of Wisconsin-Madison, United States of America

## Abstract

Cytotoxic T-lymphocytes play an important role in the protection against viral infections, which they detect through the recognition of virus-derived peptides, presented in the context of MHC class I molecules at the surface of the infected cell. The transporter associated with antigen processing (TAP) plays an essential role in MHC class I–restricted antigen presentation, as TAP imports peptides into the ER, where peptide loading of MHC class I molecules takes place. In this study, the UL49.5 proteins of the varicelloviruses bovine herpesvirus 1 (BHV-1), pseudorabies virus (PRV), and equine herpesvirus 1 and 4 (EHV-1 and EHV-4) are characterized as members of a novel class of viral immune evasion proteins. These UL49.5 proteins interfere with MHC class I antigen presentation by blocking the supply of antigenic peptides through inhibition of TAP. BHV-1, PRV, and EHV-1 recombinant viruses lacking UL49.5 no longer interfere with peptide transport. Combined with the observation that the individually expressed UL49.5 proteins block TAP as well, these data indicate that UL49.5 is the viral factor that is both necessary and sufficient to abolish TAP function during productive infection by these viruses. The mechanisms through which the UL49.5 proteins of BHV-1, PRV, EHV-1, and EHV-4 block TAP exhibit surprising diversity. BHV-1 UL49.5 targets TAP for proteasomal degradation, whereas EHV-1 and EHV-4 UL49.5 interfere with the binding of ATP to TAP. In contrast, TAP stability and ATP recruitment are not affected by PRV UL49.5, although it has the capacity to arrest the peptide transporter in a translocation-incompetent state, a property shared with the BHV-1 and EHV-1 UL49.5. Taken together, these results classify the UL49.5 gene products of BHV-1, PRV, EHV-1, and EHV-4 as members of a novel family of viral immune evasion proteins, inhibiting TAP through a variety of mechanisms.

## Author Summary

Herpesviruses have the conspicuous property that they persist for life in the infected host. This is also the case for varicelloviruses, a large subfamily of herpesviruses with representatives in humans (varicella zoster virus or VZV), cattle (bovine herpesvirus 1 or BHV-1), pigs (pseudorabies virus or PRV), and horses (equine herpesvirus or EHV type 1 and 4), among many others. Cytotoxic T-lymphocytes play an important role in the protection against viral infections, which they detect through the recognition of virus-derived peptides, presented in the context of MHC class I molecules at the surface of the infected cell. The transporter associated with antigen processing (TAP) plays an essential role in this process, as TAP imports peptides into the compartment where peptide loading of the MHC class I molecules takes place. In this study, we show that the UL49.5 proteins of BHV-1, PRV, EHV-1, and EHV-4 all block the supply of peptides through the inhibition of TAP, but that the mechanisms employed by these proteins to inhibit TAP function exhibit surprising diversity. VZV UL49.5, on the other hand, binds to TAP, but does not interfere with peptide transport. Our study classifies the UL49.5 proteins of BHV-1, PRV, EHV-1, and EHV-4 as members of a novel family of viral immune evasion proteins, inhibiting TAP through a variety of mechanisms.

## Introduction

Evolving under the selective pressure of the host immune system, herpesviruses have developed countermeasures to prevent recognition of infected cells by cytotoxic CD8^+^ T lymphocytes (CTLs). CTLs recognize viral antigens presented as peptides bound to major histocompatibility complex (MHC) class I molecules at the surface of infected cells. Herpesviruses in particular have acquired diverse mechanisms to inhibit antigen presentation in the context of MHC class I molecules, thereby escaping from elimination by CTLs [1]–[4].

Most peptides presented by MHC class I molecules are transported into the endoplasmic reticulum (ER) lumen by the transporter associated with antigen processing, TAP. TAP is a heterodimer composed of TAP1 and TAP2 subunits and belongs to the ATP-binding cassette family of transporters [5],[6]. TAP translocates peptides across the ER membrane via a conformational transition that is energized by the hydrolysis of ATP. TAP is part of the MHC class I peptide-loading complex that also contains tapasin, MHC class I heavy and light chains, and several auxiliary proteins including calreticulin and ERp57 [5], [7]–[10].

Several herpesviruses have acquired mechanisms to interfere with TAP function. Interestingly, inhibition of TAP transport is achieved through different strategies, exerted by unique gene products. Although the varicellovirus bovine herpesvirus 1 (BHV-1) and the simplexviruses herpes simplex virus type 1 and 2 (HSV-1 and -2) all belong to the subfamily of alphaherpesviruses, they block TAP through proteins that have an entirely different structure and mode of action. The inhibition of TAP by BHV-1 relies on the UL49.5 (Unique Long 49.5) gene product, a type I transmembrane protein of 75 amino acids [11]. Inactivation of TAP by UL49.5 involves two events: the arrest of the peptide transporter in a translocation-incompetent state and the proteasomal degradation of both subunits of TAP [11]. In contrast, the ICP47 proteins of HSV-1 and -2 are soluble cytosolic proteins acting as high-affinity competitors for peptide binding to TAP [12]–[18]. Within the subfamily of betaherpesviruses, human cytomegalovirus (HCMV) was found to encode a protein, US6, that inhibits TAP function by reducing the interaction of ATP with TAP [19]–[24]. The murine gammaherpesvirus-68 (MHV-68) encodes the mK3 protein that acts as a ubiquitin ligase linking MHC class I molecules and TAP to the ubiquitin/proteasome degradation pathway [25]–[35]. Recently, the BNLF2a protein of Epstein-Barr virus (EBV) and of related primate gamma-1 herpesviruses has been characterized as a potent TAP inhibitor, preventing the binding of both peptides and ATP to TAP [36].

**Figure 1:**
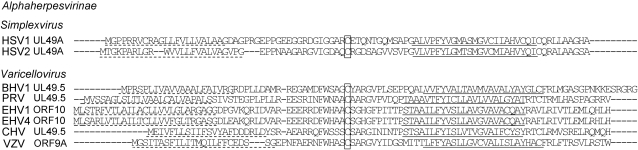
Alignment of the amino acid sequences of UL49.5 homologs of a selection of alphaherpesviruses (NCBI accession numbers are summarized in Materials and Methods). Hydrophobic residues indicative of N-terminal signal sequences (dashed line) and membrane anchor regions (bold line) are indicated. The conserved cysteine residue involved in disulfide bond formation with the viral glycoprotein M is indicated. The amino acid sequence alignment of UL49.5 homologs was performed using Vector*NTI* software (Invitrogen).

Homologs of UL49.5 (commonly known as glycoprotein N; gN) are encoded by every alpha-, beta- and gammaherpesvirus sequenced to date [37]–[39]. The UL49.5 genes are all predicted to encode a type I membrane protein with a putative cleavable signal sequence. The UL49.5 proteins interact with another herpesvirus protein, glycoprotein M (gM), with which they form a disulfide-linked heterodimer through a conserved cysteine residue within their ER-luminal/extracellular domain [37], [40]–[44]. Nevertheless, the amino acid sequences of UL49.5 proteins demonstrate considerable heterogeneity, even among varicellovirus UL49.5 proteins (Fig. 1). The only exceptions are EHV-1 and EHV-4 UL49.5, which differ by only seven amino acid residues. Thus, at this moment, it is impossible to predict on the basis of amino acid sequence whether any of these proteins have the same capacity to inhibit TAP that was found for BHV-1 UL49.5. The UL49.5 gene products of HSV-1, HSV-2, HCMV, and EBV fail to block TAP, indicating that not all UL49.5 molecules act as inhibitors of TAP [11],[37].

In this study, the effects on TAP function were assessed in more detail for UL49.5 encoded by various members of the genus *Varicellovirus*. The UL49.5 proteins of BHV-1, PRV, EHV-1, and EHV-4 were found to down-regulate MHC class I cell surface expression through TAP inhibition. Their ability to block TAP was observed in cells of the relevant host species, as well as in human cells. Using UL49.5 deletion mutants of BHV-1, PRV and EHV-1, it was shown that the UL49.5 proteins of these viruses are responsible for the inhibition of TAP-dependent peptide transport. The UL49.5 homologs of canine herpesvirus (CHV) and VZV did not affect MHC class I surface expression. BHV-1 UL49.5 strongly reduces the steady state protein levels of TAP in both bovine and human cells, whereas the UL49.5 proteins of EHV-1, EHV-4 or PRV were not observed to have this capacity. Interestingly, the EHV-1 and EHV-4 UL49.5 homologs interfere with the binding of ATP to TAP, a function that is not influenced by BHV-1 or PRV UL49.5. The UL49.5 proteins of PRV and EHV-1 arrest TAP in a translocation-incompetent state, a property that is shared with BHV-1 UL49.5. Thus, the BHV-1, PRV, EHV-1 and EHV-4-encoded UL49.5 proteins all induce a similar phenotype, i.e. inhibition of peptide transport, but their modes of action demonstrate a surprising diversity.

## Results

### Inhibition of TAP by UL49.5 proteins of varicelloviruses

**Figure 2:**
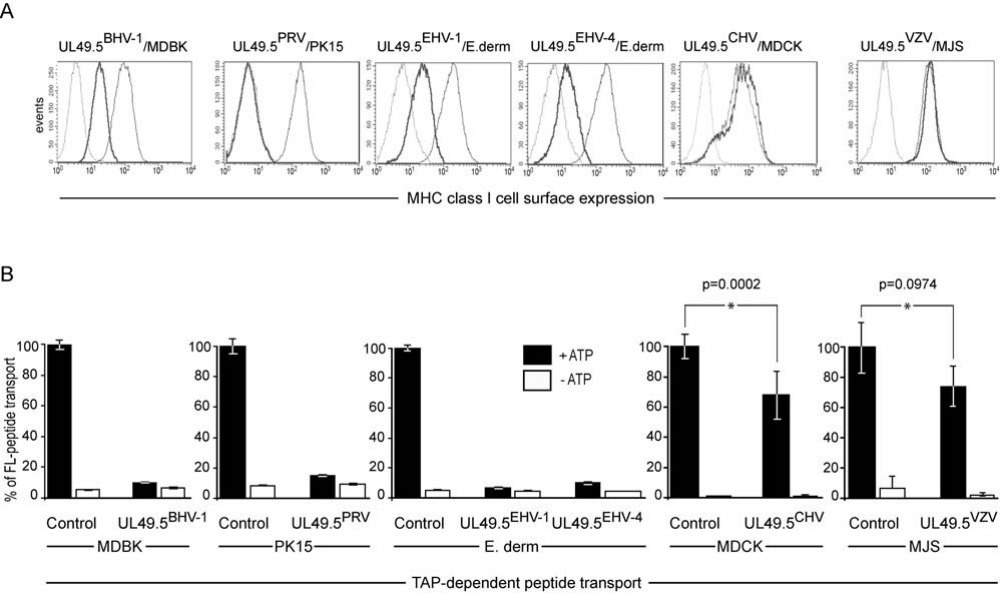
TAP inhibition by *Varicellovirus*-encoded UL49.5 homologs in natural host cells. (A) MHC class I expression in cells from the relevant host species stably expressing the UL49.5 homologs of BHV-1, PRV, EHV-1, EHV-4, CHV and VZV. Cells of bovine (MDBK), porcine (PK15), equine (E.derm), canine (MDCK), and human origin (MJS) were transduced with retroviruses expressing the respective UL49.5 proteins. Surface MHC class I molecules were stained with specific antibodies and analyzed using flow cytometry. Shown are the MHC class I levels on cells without UL49.5 (thin line) and with UL49.5 (boldface line); dashed line, goat anti-mouse phycoerythrin control. (B) TAP-dependent peptide transport is inhibited in cells from the relevant host species expressing BHV-1, PRV, EHV-1, EHV-4 and CHV UL49.5, but not in MJS cells expressing VZV UL49.5. Peptide transport was assessed in the presence and absence of ATP (black and open bars, respectively). Peptide transport is expressed as percentage of translocation, relative to the translocation observed in control cells (defined as 100%). The standard deviations are represented by the error bars. A difference at p<0.05 was considered significant.

To evaluate the TAP-inhibiting capacity of the UL49.5 proteins encoded by the varicelloviruses PRV, EHV-1, EHV-4, CHV and VZV, cell lines of the relevant host species were transduced using a retrovirus-based gene delivery system to express the corresponding UL49.5 proteins. Down-regulation of MHC class I expression by the UL49.5 gene products was evaluated using flow cytometry. In cells expressing UL49.5 of BHV-1, PRV, EHV-1 and EHV-4, MHC class I surface expression was reduced (Fig. 2A). The UL49.5 proteins of CHV and VZV failed to down-regulate MHC class I surface expression. These results indicate that UL49.5 of BHV-1, PRV, EHV-1 and EHV-4 interfere with MHC class I-restricted antigen presentation.

To investigate whether the observed down-regulation of MHC class I cell surface expression relies on the inhibition of TAP, species-specific cell lines stably expressing these UL49.5 homologs were evaluated for TAP-dependent peptide transport. The UL49.5 proteins of BHV-1, PRV, EHV-1 and EHV-4 strongly inhibited TAP activity in the corresponding natural host cell lines (Fig. 2B). Despite the absence of a detectable reduction in cell surface MHC class I levels (Fig. 2A), some inhibition of TAP-dependent peptide transport was observed in canine cells expressing the CHV UL49.5 protein (Fig. 2B). Apparently, the inhibition of TAP by CHV UL49.5 was insufficient to observe MHC class I downregulation at the cell surface. VZV UL49.5 had no significant effect on TAP activity. Thus, although the amino acid sequences of the UL49.5 proteins of BHV-1, PRV, and EHV-1/EHV-4 demonstrate considerable variation (Fig. 1), their ability to inhibit TAP was found to be a common property of these varicellovirus gene products.

### The interaction of VZV UL49.5 with the peptide-loading complex has no functional consequences for MHC class I-restricted antigen presentation

**Figure 3:**
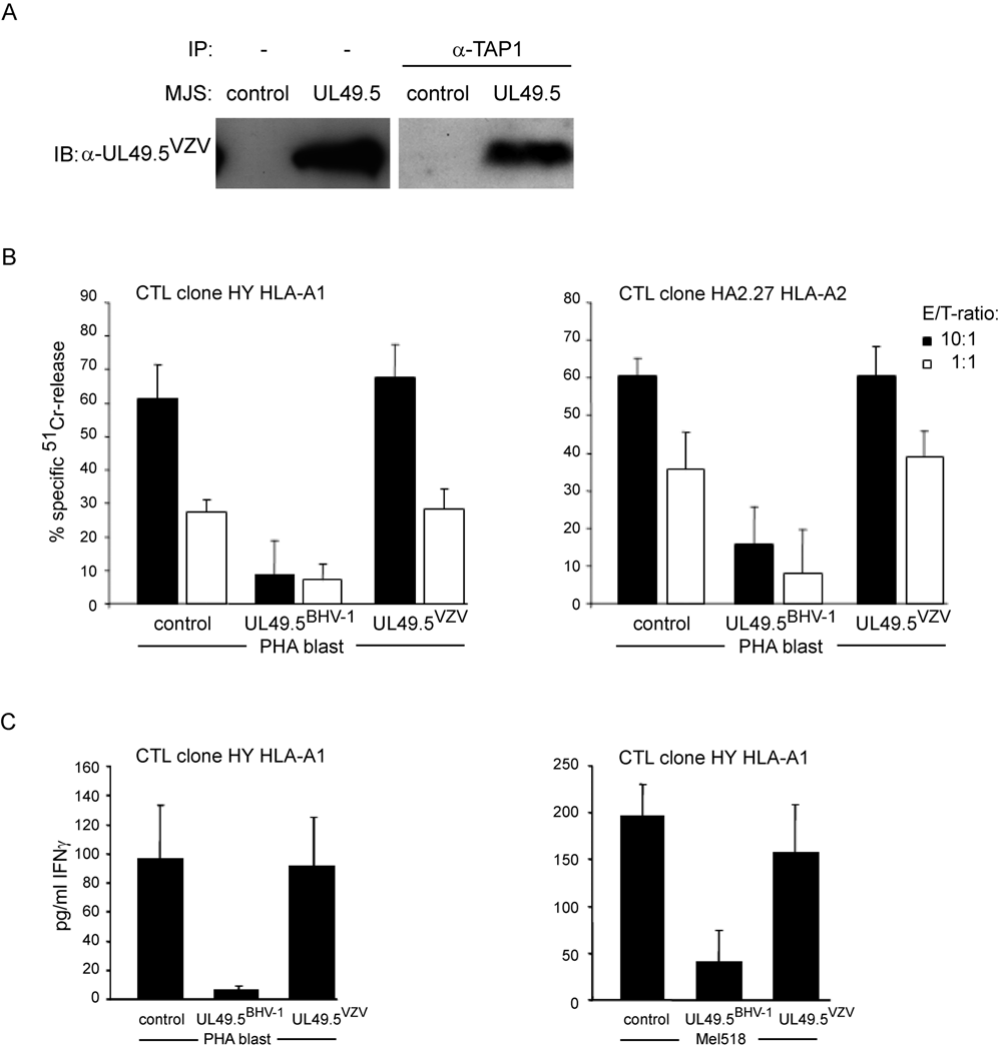
VZV UL49.5 interacts with the peptide-loading complex, but does not inhibit MHC class I-restricted T cell recognition. (A) TAP1 was immunoprecipitated (IP) from MJS cells expressing EHV-1 or VZV UL49.5 proteins. Co-precipitating UL49.5 proteins were analyzed by immunoblotting (IB) using antibodies against EHV-1 or VZV UL49.5. Left panel: cell lysates were loaded on SDS-PAGE directly and stained by immunoblotting. (B) BHV-1 or VZV UL49.5-expressing PHA-treated T-cell blasts were labeled with ^51^Cr and used as target cells for CTL clones specific for the minor histocompatibility antigens HY and HA-2, recognized in the context of HLA-A1 and HLA-A2, respectively. Specific lysis was determined by measuring ^51^Cr release from the target cells after 4 hrs. Effector/target (E/T) ratios are indicated. (C) PHA-treated T-cell blasts or a melanoma cell line (Mel518) carrying the HY antigen were incubated with the HY-specific CTL clone HY HLA-A1. IFN-γ levels released by the CTL clones were determined from the supernatants of the co-cultures after 24 hrs.

VZV infection has been shown to cause down-regulation of MHC class I expression at the cell surface [45]–[47]. This phenotype could not be reproduced by the VZV-encoded UL49.5 protein when expressed individually (Fig. 2A). To examine whether the absence of MHC class I down-regulation by VZV UL49.5 is due to a loss of the interaction of the viral protein with the TAP complex, TAP was immunoprecipitated from VZV UL49.5-expressing MJS cells that were solubilized in the presence of the mild detergent digitonin. The resulting protein complexes were separated by SDS PAGE and analyzed for the presence of UL49.5 by immunoblotting. Surprisingly, VZV UL49.5 was found to interact with the TAP complex (Fig. 3A).

Although VZV UL49.5 associates with TAP, this appears to be insufficient to inhibit peptide transport effectively (Fig. 2A and B). VZV UL49.5 could, however, interfere with peptide-loading and MHC class I-restricted antigen presentation in a different way. For instance, the US3 protein encoded by human cytomegalovirus binds both tapasin and TAP, without having an effect on TAP function. Instead, US3 impairs tapasin-dependent peptide loading and optimization of the MHC class I peptide cargo [48],[49]. To investigate whether VZV UL49.5 inhibits MHC class I-mediated antigen presentation via a mechanism similar to that of US3, functional T cell assays were performed using a panel of human leukocyte antigen (HLA)-A1 and HLA-A2-restricted CTL clones. It is known that especially HLA-A1-restricted peptide presentation strongly depends on the function of tapasin. Antigen-presenting phytohemagglutinin (PHA)-treated T cell blasts and the melanoma cell-line Mel518 were transduced to express the UL49.5 proteins of VZV and BHV-1. While the presence of BHV-1 UL49.5 greatly reduced specific lysis of the PHA-blasts by CTLs, the expression of VZV UL49.5 had no detectable effect (Fig. 3B). This was observed for HLA-A1 and HLA-A2-restricted CTL clones. VZV UL49.5-expressing and control target cells induced IFNγ production by the CTLs, while reduced IFNγ production was observed when BHV-1 UL49.5 was expressed by the target cells (Fig. 3C). This reflects effective inhibition of CTL recognition by BHV-1 but not VZV UL49.5. Thus, despite the interaction of the VZV UL49.5 protein with the peptide-loading complex, no interference with MHC class I-restricted antigen presentation could be detected.

### UL49.5 is responsible for the inhibition of TAP in virus-infected cells

**Figure 4:**
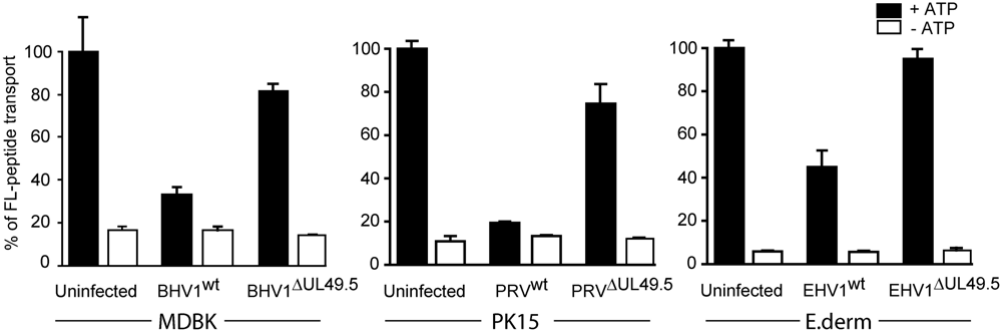
UL49.5 is responsible for TAP-inhibition in virus-infected natural host cells. Bovine cells (MDBK), porcine cells (PK15), and equine cells (E.derm) were infected with wild type BHV-1, PRV, or EHV-1, respectively, or with the corresponding UL49.5-negative recombinant viruses. In all experiments, mock-treated (uninfected) cells from the relevant host species were used as a control. Peptide transport was assessed at 5 hrs post-infection in the presence and absence of ATP (black and open bars, respectively). The data are expressed as percentage of translocation, relative to the translocation observed in control cells (defined as 100%).

Having observed that the UL49.5 proteins of BHV-1, PRV, EHV-1, and EHV-4 interfere with MHC class I-restricted antigen presentation when expressed individually, we next investigated whether the various UL49.5 proteins are responsible for TAP inhibition during infection with BHV-1, PRV, or EHV-1. Peptide transport activity was examined in natural host cells infected with wild type viruses or with the corresponding recombinant viruses lacking a functional UL49.5 gene [41],[43]. Whereas the wild-type viruses effectively blocked peptide transport, this inhibition was not observed in cells infected with the mutant viruses lacking UL49.5 (Fig. 4). These findings indicate that during infection with BHV-1, PRV and EHV-1, the UL49.5 gene products of these viruses are responsible for the inhibition of peptide translocation by TAP observed in virus-infected cells previously [50]–[52].

### UL49.5 protein of BHV-1 but not of PRV or EHV-1 reduces TAP1 and TAP2 steady state levels

**Figure 5:**
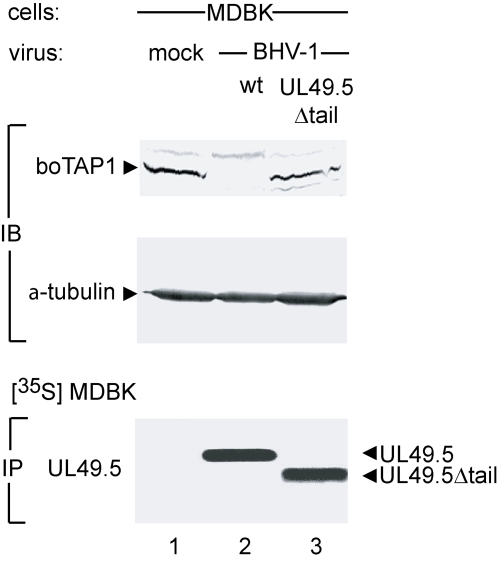
Degradation of bovine TAP is mediated by BHV-1UL49.5 through its cytoplasmic tail. MDBK cells were mock-infected or infected with wild type BHV-1 or BHV-1 expressing UL49.5Δtail for 12 hours. The levels of bovine TAP1 and, as a control, α-tubulin were assessed in lysates of infected cells by immunoblotting (IB). BHV-1 UL49.5 was immunoprecipitated from lysates of metabolically labeled cells (IP).

Next, the mechanism of TAP inhibition by the various UL49.5 proteins was investigated. Expression of BHV-1 UL49.5 strongly reduced TAP1 and TAP2 protein levels in human MJS cells [11]. It was shown that the cytoplasmic domain of UL49.5 is required for mediating proteasome-dependent degradation of TAP. To investigate whether BHV-1 UL49.5 has a similar mode of action in natural host cells, bovine MDBK cells were infected with wild type BHV-1 or a recombinant virus expressing a UL49.5 protein that lacks its cytoplasmic domain (UL49.5Δtail). Steady state protein levels of bovine TAP were evaluated by immunoblotting. Whereas bovine TAP was readily detectable in uninfected MDBK cells, it was no longer observed in cell lysates from wild-type BHV-1 infected cells (Fig. 5; upper panel, compare lanes 1 and 2). Interestingly, in cells infected with the recombinant virus expressing the UL49.5Δtail mutant, TAP1 steady state levels were not affected (compare lanes 2 and 3). As a control, α-tubulin was consistently detected in all samples (Fig. 5, middle panel). Immunoprecipitation of UL49.5 from the infected cells confirmed the expression of the wild-type and recombinant proteins (Fig. 5; lower panel). These findings indicate that the degradation of TAP by UL49.5 previously observed in human cells also occurs in bovine cells. In addition, like in human cells, the cytoplasmic domain of UL49.5 is critical to TAP degradation in the natural host cells.

**Figure 6:**
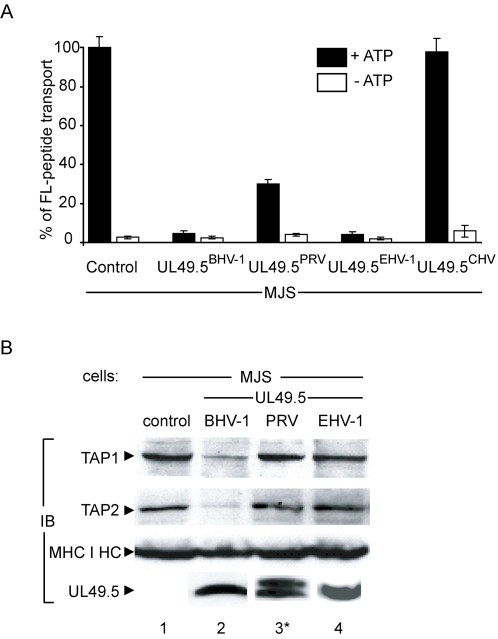
UL49.5 of BHV-1 but not of PRV or EHV-1 reduces TAP1 and TAP2 steady state levels in human cells. (A) TAP-dependent peptide transport is inhibited in human melanoma cells (MJS) stably expressing the UL49.5 homologs of BHV-1, PRV and EHV-1, but not CHV. Peptide transport is depicted as percentage of translocation, relative to the translocation observed in control cells (defined as 100%). (B) Steady state protein levels of TAP1, TAP2, MHC class I heavy chains (MHC I HC), and UL49.5 in control cells and cell lines expressing UL49.5 of BHV-1, PRV and EHV-1. Proteins present in post-nuclear supernatants were separated using SDS-PAGE and detected by immunoblotting (IB) using antibodies specific for TAP1, TAP2, MHC class I heavy chains, and the UL49.5 proteins of BHV-1, PRV and EHV-1. *The doublet of PRV UL49.5 is probably related to differential glycosylation.

To further address the molecular basis of TAP inhibition mediated by PRV, EHV-1, EHV-4, and CHV UL49.5, these proteins were stably expressed in human melanoma (MJS) cells. Like BHV-1 UL49.5, the PRV and EHV-1 UL49.5 proteins were capable of blocking human TAP (Fig. 6A). CHV UL49.5 did not inhibit peptide transport in human cells, while in canine cells some reduction in TAP activity was observed without a reduction of MHC class I surface expression (Fig. 2A and B). Expression of BHV-1 UL49.5 in MJS cells resulted in reduced TAP1 and TAP2 protein levels, which is in accordance with previous observations (Fig. 6B; compare lanes 1 and 2) [11]. In contrast, expression of the UL49.5 homologs of PRV and EHV-1 did not affect TAP1 and TAP2 steady state levels in MJS cells (Fig. 6B; lanes 3 and 4). These findings indicate that the UL49.5 homologs of PRV and EHV-1 inhibit peptide transport by TAP through a different mechanism than by mediating degradation of TAP.

### Peptide binding to TAP is not affected by EHV-1 and PRV UL49.5 proteins

**Figure 7:**
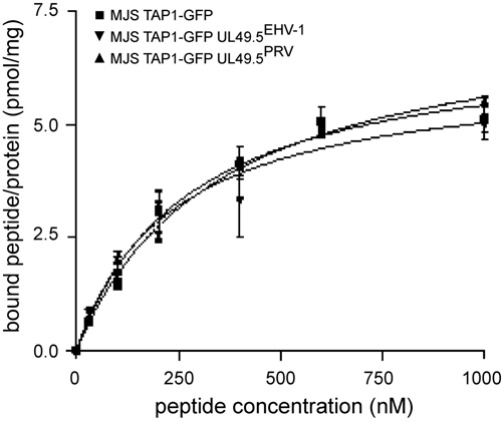
UL49.5 of PRV and EHV-1 do not interfere with peptide binding to TAP. To evaluate peptide binding to the TAP complex, microsomal membranes from MJS TAP1-GFP cells (control; ▪), or MJS TAP1-GFP cells expressing UL49.5 of PRV (▴) or EHV-1 (▾) were incubated with increasing concentrations of the radiolabeled peptide (RR[^125^I]YQKSTEL). Unspecific binding was determined in the presence of 200-fold excess of ICP47 (data not shown). The amount of specifically bound peptide per amount of microsomal protein is plotted against the peptide concentration. K_d_ values for control MJS: 277±58 nM, for MJS UL49.5^PRV^: 351±42 nM and for MJS UL49.5^EHV-1^: 236±71 nM.

The translocation of peptides into the ER lumen is initiated by the association of peptides with the peptide-binding site of TAP [53]. To investigate whether the inhibition of peptide transport by PRV and EHV-1 UL49.5 involves blocking of peptide binding to TAP, microsomes were isolated from MJS cells expressing PRV and EHV-1 UL49.5. Microsomes were incubated with a ^125^I-labeled reporter peptide (Fig. 7). At all concentrations tested, the peptide-binding capacity (Bmax) was similar for microsomes prepared from control cells and from cells expressing the PRV or EHV-1 UL49.5 proteins. Most importantly, the binding affinity (K_d_) for the peptides was not changed by the viral inhibitors, demonstrating preservation of the peptide binding site of the TAP complex. This has also been observed for BHV-1 UL49.5 [11] and indicates that inhibition of TAP-mediated peptide transport by the BHV-1, PRV, and EHV-1 UL49.5 proteins does not rely on interference with peptide binding.

### EHV-1 UL49.5 inhibits ATP-binding by TAP

**Figure 8:**
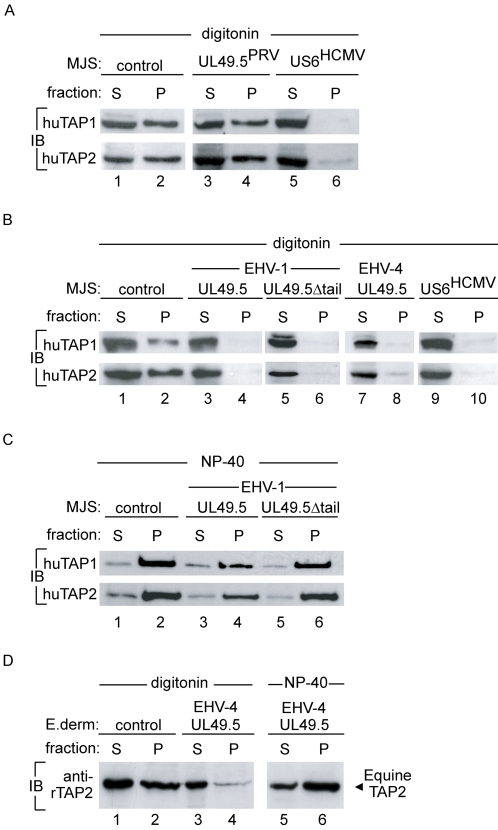
UL49.5 of EHV-1 and EHV-4 block ATP binding to human and equine TAP. (A, B, C) MJS cells expressing the UL49.5 proteins or the HCMV-derived TAP-inhibitor US6 and (D) E.derm cells expressing EHV-4 UL49.5 were lysed using digitonin or NP40 as indicated. Post nuclear lysates were incubated with ATP-agarose. The pellet (P) contains the ATP-binding proteins. The supernatant (S) contains proteins incapable of binding ATP. ATP-bound (ATP-agarose beads; pellet) and unbound (soluble; supernatant) fractions were separated by centrifugation and analyzed using SDS-PAGE and immunoblotting (IB) with antibodies against the proteins indicated.

Since ATP-binding and hydrolysis are required to energize peptide translocation by TAP [54]–[56], it was investigated whether the expression of the PRV, EHV-1, and EHV-4 UL49.5 proteins affected binding of ATP to TAP. Previous experiments indicated that the BHV-1 UL49.5 protein did not influence the interaction of ATP with TAP [11]. The ATP-binding capacity of TAP in lysates from MJS cells (control) was compared to the binding in lysates from MJS cells stably expressing UL49.5 of PRV, EHV-1 or EHV-4, or the HCMV-encoded US6 protein. US6 is known to strongly inhibit ATP-binding to TAP [22],[24]. Cell lysates prepared in the presence of the mild detergent digitonin were incubated with ATP-agarose beads. Proteins bound to the ATP-agarose (Fig. 8; pellet “P”) were eluted from the beads with EDTA and displayed next to the unbound supernatant fractions (Fig. 8; “S”). TAP1 and TAP2 were detected by immunoblotting.

PRV UL49.5 did not alter the binding of ATP to TAP1 or TAP2 (Fig. 8A; compare lanes 2 and 4). As expected, the expression of US6 completely abolished the interaction of ATP with TAP (Fig. 8A; lane 6). These data show that TAP retains the capacity to bind ATP in the presence of PRV UL49.5.

In EHV-1 and EHV-4 UL49.5-expressing cells, neither TAP1 nor TAP2 could be detected in the ATP-agarose fraction (Fig. 8B; compare lane 2 with lanes 4 and 8). Since the C-terminus of UL49.5 is exposed in the cytosol, this domain might be responsible for the inhibition of ATP-binding to the nucleotide-binding domains of TAP. To evaluate whether the C-terminus of UL49.5 blocks ATP-binding to TAP, a truncated form of EHV-1 UL49.5 lacking the cytoplasmic domain was constructed and expressed in MJS cells. The EHV-1 UL49.5Δtail recombinant still interfered with ATP-binding to TAP (Fig. 8B; lane 6). When the association of wild type or mutant EHV-1 UL49.5 with TAP was disrupted by lysis of the cells in NP-40, the ability of TAP1 and TAP2 to bind to the ATP-agarose was restored (Fig. 8C, lanes 4 and 6; also compare Fig. 8B lanes 4 and 6 with Fig. 8C lanes 4 and 6, respectively). These results indicate that the EHV-1 UL49.5 protein is capable of interfering with the recruitment of ATP by human TAP independent of the cytoplasmic domain of UL49.5.

The ability of EHV-1 and EHV-4 UL49.5 to interfere with the binding of ATP to equine TAP was assessed in E. derm cells (data shown for EHV-4). Like human TAP (Fig. 8B lane 8), equine TAP2 was not able to bind ATP-agarose in the presence of EHV-4 UL49.5 (Fig. 8D; compare lanes 2 and 4). When the experiment was performed in the presence of NP-40, the ability of equine TAP2 to bind ATP was restored (Fig. 8D; compare lanes 4 and 6). These results indicate that the UL49.5 proteins of EHV-1 and EHV-4 inhibit human and equine TAP through similar mechanisms, rendering both human and equine TAP molecules incapable of recruiting ATP.

### UL49.5 proteins arrest TAP in a translocation-incompetent state

**Figure 9:**
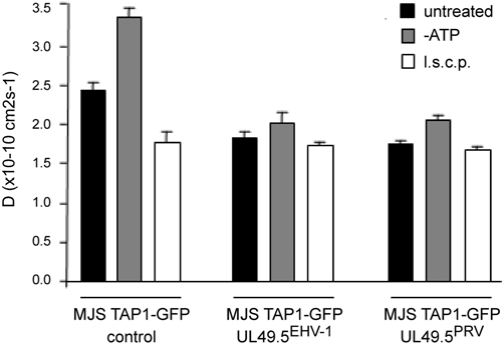
UL49.5 homologs arrest TAP in a translocation-incompetent state. The lateral mobility of the TAP complex was analyzed in MJS TAP1-GFP cells using confocal microscopy and FRAP. A circular spot in the ER was bleached and recovery of fluorescence was monitored. The half-time for recovery was determined and used to calculate the diffusion coefficient D. Where indicated, ATP was depleted (-ATP) or saturating amounts of substrate peptides (long side chain peptides, l.s.c.p.) were micro-injected into the cells.

To obtain further insight into the strategies used by EHV-1 and PRV UL49.5 to block TAP transport, Fluorescence Recovery After Photobleaching (FRAP) assays were performed. With this technique, conformational changes of TAP that occur during peptide translocation can be indirectly visualized by measuring the lateral mobility of green fluorescence protein (GFP)-tagged TAP within the ER membrane. It has been shown that the lateral mobility of TAP is inversely proportional to its activity, as peptide-transporting TAP molecules diffuse at a slower rate than inactive, closed TAP complexes [57]. In the absence of ATP, the translocation cycle cannot be initiated and consequently TAP will have a closed, more compact conformation. In agreement with this, depletion of ATP results in increased mobility of TAP in the ER membrane (Fig. 9; control samples, compare black and grey bars). The complex can be trapped in the active conformation by adding long side chain peptides (l.s.c.p.). These peptides bind to TAP, but cannot be translocated over the ER membrane, which results in a retained open conformation and therefore a slow diffusion rate of TAP in the ER membrane [57] (Fig. 9; control samples, white bar).

Expression of EHV-1 and PRV UL49.5 results in a decreased mobility of TAP (Fig. 9; compare untreated samples/black bars). Whereas the diffusion rate of TAP increased considerably in ATP-depleted control cells, only a slight increase in TAP mobility was detected upon ATP depletion in the UL49.5-expressing cells (grey bars). The failure of TAP to respond to ATP depletion in the EHV-1 UL49.5 cells is in agreement with the observation that this protein interferes with ATP binding to TAP (Fig. 8B). Although ATP can still bind to TAP in the presence of PRV UL49.5 (Fig. 8A), ATP depletion induces only a minor change in TAP mobility in the PRV UL49.5 cells (Fig. 9). Apparently, the presence of PRV UL49.5 prohibits conformational transitions that normally follow ATP-binding.

In the presence of the UL49.5 proteins, l.s.c.p. were also unable to induce conformational changes within the TAP complex (Fig. 9). Since peptides can still bind to TAP in the presence of EHV-1 and PRV UL49.5 (Fig. 7), the failure of l.s.c.p. to induce conformational changes again suggests that the UL49.5 proteins arrest TAP in a translocation-incompetent state.

## Discussion

This study identifies the UL49.5 proteins of BHV-1, PRV, EHV-1, and EHV-4 as members of a novel class of viral immune evasion proteins. The UL49.5 gene products interfere with MHC class I antigen presentation by blocking the supply of antigenic peptides in the ER lumen through inhibition of TAP. Within the UL49.5 family of TAP inhibitors, heterogeneity is observed with respect to the mechanisms that underlie TAP inhibition. Whereas BHV-1 UL49.5 targets TAP for proteasomal degradation [11], PRV and EHV-1 UL49.5 do not diminish the steady state levels of TAP1 or TAP2. Interestingly, EHV-1 and EHV-4 UL49.5 interfere with the binding of ATP to TAP, a function that is not influenced by BHV-1 or PRV UL49.5. All TAP-inhibiting UL49.5 proteins arrest the transporter complex in a translocation-incompetent state.

**Figure 10:**
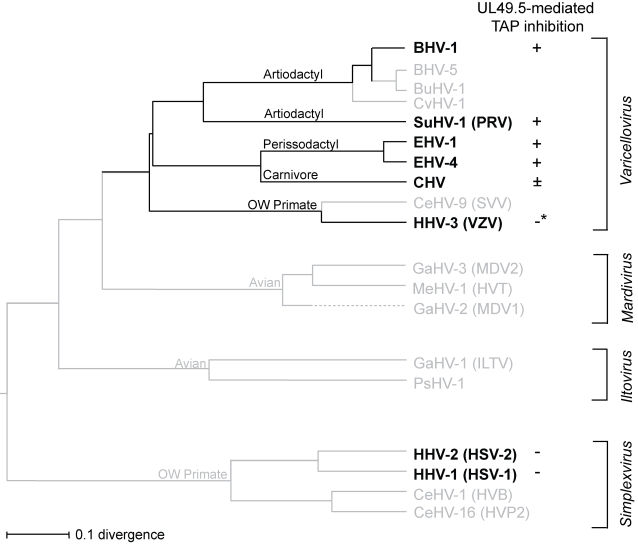
Phylogenetic tree of alphaherpesvirus UL49.5 proteins constructed on the basis of all presently known UL49.5 amino acid sequences of *Alphaherpesvirinae* using ClustalV. The ClustalV (PAM250) method was used under the default settings of the alignment program MegAlignTM 500 of the sequence analysis software DNA* of DNASTAR Inc. The UL49.5 proteins for which TAP inhibition has been tested are shown in bold. *The HHV-3 (VZV) UL49.5 protein binds TAP, but does not block peptide transport. The NCBI accession numbers are provided in Materials and Methods.

UL49.5 homologs are encoded by all *Herpesviridae* analyzed to date [38]. However, the TAP-inhibiting capacities of these proteins appear to be restricted to certain members of the genus *Varicellovirus*. Members of this virus genus have co-evolved with their respective host species [39]. Viruses of even-toed ungulates or *Artiodactyla* like BHV-1 and PRV co-evolved with cattle and pigs; viruses of odd-toed ungulates or *Perissodactyla* (EHV-1 and EHV-4) with horses; the carnivore viruses FHV-1 and CHV with cats and dogs, and the Old World primate virus VZV with humans [39] (Fig. 10). The identification of the UL49.5 proteins encoded by BHV-1, PRV, EHV-1, and EHV-4 as members of the UL49.5 family of TAP inhibitors suggests that more UL49.5 proteins with this property may be found in varicelloviruses of even- and odd-toed ungulate hosts. Considering the shared evolution of (herpesviruses from) carnivores and (herpesviruses from) odd-toed ungulates [39], CHV UL49.5 was expected to inhibit TAP as effectively as EHV-1 and EHV-4 UL49.5. However, the reduction of TAP-dependent peptide transport caused by CHV UL49.5 was very moderate compared to the inhibition by the other TAP-inhibiting UL49.5 proteins. The identification of the UL49.5 domains contributing to TAP inhibition will provide more insights into these differences.

VZV infection of human cells results in reduced expression of MHC class I at the cells surface [45]–[47]. The VZV ORF66-encoded serine-threonine protein kinase has been shown to be one of the VZV proteins contributing to MHC class I down-regulation in VZV-infected cells [47]. However, a VZV recombinant lacking a functional ORF66 product still causes down-regulation of MHC class I surface expression, indicating that additional modulators of MHC class I-restricted antigen presentation are encoded by VZV. The observed down-regulation of MHC class I surface expression on VZV-infected cells [45]–[47] is not induced by UL49.5 when expressed individually. Despite the observed interaction between VZV UL49.5 and the peptide-loading complex, this protein alone did not block peptide transport by TAP and it had no effect on antigen recognition by HLA-A1 and HLA-A2-restricted CTL clones. As VZV occupies a somewhat isolated position in the phylogenetic tree of varicelloviruses (Fig. 10), it seems likely that evolutionary divergence has influenced VZV to acquire a separate mechanism to interfere with MHC class I-restricted antigen presentation. Alternatively, UL49.5 might co-operate with another unidentified VZV-encoded protein in order to reduce antigen presentation by MHC class I molecules. During virus infection, UL49.5 can be found in a complex with glycoprotein M (gM). However, the co-expression of VZV UL49.5 and glycoprotein M has no effect on the expression of MHC class I molecules at the cell surface [47], indicating that gM does not act as a modulator of UL49.5 with respect to TAP inhibition.

Interaction with the conserved viral membrane glycoprotein M appears to be a common property of all UL49.5 homologs, as is the presence of a single cysteine residue in their ER-luminal/extracellular domain [41]–[44]. This cysteine residue is involved in the interaction of UL49.5 with gM, with which it forms a disulfide-linked heterodimers [37],[40]. The complex of UL49.5 and gM is implicated in virion maturation and membrane fusion processes [58],[59]. Interestingly, the interaction of BHV-1 UL49.5 with gM interferes with its capacity to block TAP [42]. Nevertheless, UL49.5 blocks peptide transport by TAP in BHV-1-infected cells. This may be explained by the fact that UL49.5 is expressed prior to and in excess of the early-late gM [42].

Interference with TAP-mediated peptide transport is an effective way of reducing CTL recognition and is used by several other herpesviruses, including HSV-1 and -2, HCMV, MHV-68, and EBV [12]–[36]. Compared to the other herpesvirus-encoded TAP inhibitors, the cross-species activity of UL49.5 proteins is remarkable. Except for CHV UL49.5, the UL49.5 proteins of BHV-1, PRV, EHV-1, and EHV-4 all exhibit the ability to target human TAP. In addition, BHV-1 UL49.5 inhibits peptide transport by murine [60], rat, equine, and porcine TAP (D.K.L. and M.V., unpublished observations). Human, porcine, bovine, and rodent TAP1 and TAP2 demonstrate a substantial degree of amino acid identity (70–80%) [61]. Thus, the ability of UL49.5 proteins to act across species barriers most likely relies on structural homology within the TAP domains critically involved in UL49.5-TAP interaction. Apparently, this is less so for the domains within TAP that are targeted by US6, mK3 and BNLF2a, whose actions seem to be restricted largely to the natural host species. BHV-1 UL49.5 reduces TAP protein levels in bovine, human, and murine cells, and also mediates degradation of human TAP in insect cells when co-expressed with UL49.5 [11],[60],[62], indicating conservation of the pathway involved in this degradation process.

The UL49.5 proteins exhibit unexpected differences in their mechanisms of TAP inhibition, despite their close evolutionary relatedness. The cytoplasmic domain of BHV-1 UL49.5 is essential for mediating degradation of both human and bovine TAP. EHV-1 and PRV UL49.5 have no influence on the stability of TAP. Apparently, degradation of TAP is facilitated by a yet unknown signal within the C-terminal domain of BHV-1 UL49.5, which is not present in the other homologs. Studies to identify the nature of this sequence motif are in progress.

The interaction of EHV-1 and EHV-4 UL49.5 with TAP blocks ATP binding to TAP. This feature distinguishes EHV UL49.5 from the other homologs studied. Interestingly, removal of the cytoplasmic domain of the EHV-1 UL49.5 protein did not restore the ability of TAP to bind ATP. Therefore, a direct interaction of EHV-1 UL49.5 with the cytosolic nucleotide binding domains of TAP is unlikely. Instead, the viral protein appears to arrest TAP in a translocation-incompetent state, incompatible with ATP-binding. This may resemble the type of structural change caused by HCMV US6 [23],[63]. US6, a type I transmembrane protein, interacts with the luminal side of the TAP transporter and blocks ATP-binding by prohibiting essential conformational rearrangements within TAP. The inability of the BHV-1 and PRV UL49.5 homologs to interfere with ATP-binding could be due to a slightly different conformational change induced by these proteins.

Based on the results presented in this study, the UL49.5 proteins encoded by BHV-1, PRV, EHV-1, and EHV-4 can be classified as a new family of TAP-inhibiting proteins. These proteins share the ability of inducing a conformational arrest of TAP, which results in impaired peptide transport and inhibition of MHC class I-restricted antigen presentation. In view of these joint features it is likely that the TAP inhibiting UL49.5 proteins originate from a common ancestral protein, which acquired this capacity earlier during evolution. The VZV UL49.5 protein may be a rudimentary form with respect to TAP inhibition, or it may have lost its TAP inhibitory capacity later on. Alternatively, it may require additional VZV proteins for the inhibition of TAP.

This study has revealed unexpected variation among UL49.5 proteins of varicelloviruses with respect to their mechanisms of TAP inhibition. These differences can be related to distinct evolutionary pathways of these varicelloviruses. The UL49.5 family of TAP-inhibiting proteins does not demonstrate any structural or functional similarity to TAP-inhibiting proteins encoded by other herpesviruses, for instance ICP47, US6, mK3, or BNLF2a. This diversity of TAP-inhibiting proteins acquired by distantly related members of the subgroups of alpha-, beta-, and gammaherpesviruses is remarkable and presents a striking example of functional convergent evolution. At the same time, this identifies TAP as an Achilles' heel of the MHC class I antigen presentation pathway. Inhibition of TAP has apparently provided a strong advantage to these herpesviruses during co-evolution with their hosts.

## Materials and Methods

### UL49.5 constructs

Purified viral DNA from BHV-1 strain Lam and CHV strain Eva (Animal Sciences Group, Lelystad, The Netherlands), PRV strain Kaplan [41], EHV-1 strain Ab-4 (kindly provided by J. Rola; National Veterinary Research Institute, Pulawy, Poland) and EHV-4 (kindly provided by R. de Groot; Dept. of Infectious Diseases and Immunology, Utrecht University, The Netherlands), and VZV (viral DNA extracted from patient material; kindly provided by E. Klaas, Leiden University Medical Center, Leiden, The Netherlands) were used as a template for polymerase chain reaction (PCR) amplification. PCR-reactions were performed under standard conditions using *Pfu* DNA polymerase (Invitrogen) and specific primers (Table 1) for amplification of the full length coding sequence of the UL49.5 genes of BHV-1 [11], PRV, EHV-1, EHV-4, CHV and VZV UL49.5. The sequences of the primers are based on published sequences found in the NCBI database, except for the sequence of the CHV primers (Haanes, E. and Rexann, F. ‘Recombinant canine herpesviruses’, patent number EPO910406, publication date 1997-08-21). To generate the EHV-1 UL49.5Δtail construct, primers (Table 1) were used to obtain a PCR product lacking 3′-terminal 45 nucleotides, thereby deleting the 15 carboxy-terminal amino acids. PCR-generated products were sequenced and inserted into the retroviral expression vectors pLZRS-IRES-GFP or pLZRS-IRES-ΔNGFR, upstream of the internal ribosome entry site (IRES) element. pLZRS vector information can be obtained at www.stanford.edu/group/nolan/retroviral_systems/retsys.html).

**Table 1:** PCR primers used

Primer name	Primer sequence (restriction enzymes used in bold)
PRV UL49.5	Fw: 5′-CGC**GGATCC**GACACACCAGGATGGTC-3′
PRV UL49.5	Rev: 5′-GCG**GAATTC**GGATCGCTCTTTATACGC-3′
EHV-1UL49.5	Fw: 5′-GCCGCCACCATGCTGTCCACGAGATTC-3′
EHV-1UL49.5	Rev: 5′-TTGTCAATGCAGGTGTTGCAACATCTC-3′
EHV-1UL49.5Δtail	Fw: 5′-GA**GAATTC**AGGACCATGCTGTCCACGAGATTC-3′
EHV-1UL49.5Δtail	Rev: 5′-CG**CTCGAG**CTTCAGCGGTATGCCTG-3′
EHV-4 UL49.5	Fw: 5′-GCCGCCACCATGTTGTCAGCGAGATTAG-3′
EHV-4 UL49.5	Rev: 5′-TGCTCAGTGTAGGTGTCGCAAATC-3′
CHV UL49.5	Fw: 5′ -GCCGCCACCATGGAGATAGTATTTTTAC-3′
CHV UL49.5	Rev: 5′ -CCATTAGTGTTGCATCTGACGAAGTTC-3′
VZV UL49.5	Fw: 5′-GCC**GGATCC**AAGATGGGATCAATTAC-3′
VZV UL49.5	Rev: 5′-CCG**GAATTC**CGGTTACCACGTGCTGCG-3′

### Cell Lines and Retroviral Transduction

The human melanoma cell line Mel JuSo (MJS), MJS TAP1-GFP [57] and Madin-Darby bovine kidney (MDBK) cells (American Type Culture Collection, ATCC) were maintained in RPMI-1640 medium; GP2-293 cells, porcine kidney (PK15) cells, the embryonic bovine trachea (EBTr) cell line, Madin-Darby canine kidney I (MDCK I) cells, and the equine epithelial cell line E.derm were maintained in DMEM medium. Media were supplemented with 10% heat-inactivated fetal bovine serum (FBS) (with the exception of E.derm cells that required 20%), 2 mM L-glutamine (Invitrogen), 140 IU/ml penicillin and 140 µg/ml streptomycin. PHA-treated T-cell blasts positive for HLA-A1 and HLA-A2 were generated from PBMCs by stimulation with 0.8 µg/ml PHA and were subsequently cultured in IMDM supplemented with 100 IU/ml IL-2 and 10% FBS. The HLA-A2-expressing melanoma cell line 518, Mel518 (a kind gift from E. Verdegaal, department of Clinical Oncology, Leiden University Medical Center, Leiden, The Netherlands) was maintained in DMEM containing 4.5 mM glucose, supplemented with 8% FBS, 2 mM L-glutamine (Invitrogen), 140 IU/ml penicillin and 140 µg/ml streptomycin.

Recombinant retroviruses were prepared using the Phoenix amphotropic packaging system as described previously (www.stanford.edu/group/nolan/retroviral_systems/retsys.html ). MJS, MDCK I, PK15, and E.derm cells were transduced with recombinant retroviruses to generate the following stable cell lines: MJS, MDCK I, PK15, and E.derm controls (containing BHV-1 UL49.5 in the anti-sense orientation, GFP^+^); MJS UL49.5^BHV-1^, PHA T-cell blast UL49.5^BHV-1^ and Mel518 UL49.5^BHV-1^ (containing BHV-1 UL49.5 in the **s**ense **o**rientation (SO), GFP^+^); MJS UL49.5^VZV^, PHA T-cell blast UL49.5^VZV^ and Mel518 UL49.5^VZV^ (containing VZV UL49.5 SO, GFP^+^); MJS UL49.5^CHV^ and MDCK I UL49.5^CHV^ (containing CHV UL49.5 SO, GFP^+^); MJS UL49.5^PRV^ and PK15 UL49.5^PRV^ (containing PRV UL49.5 SO, GFP^+^); MJS UL49.5^EHV-1^ and E.derm UL49.5^EHV-1^ (containing EHV-1 UL49.5 SO, GFP^+^); MJS UL49.5^EHV-1Δtail^ (containing tail-less EHV-1 UL49.5 SO, GFP^+^); MJS UL49.5^EHV-4^ and E.derm UL49.5^EHV-4^ (containing EHV-4 UL49.5 SO, GFP^+^). In addition, MJS TAP1-GFP cells were transduced with recombinant retrovirus to generate MJS TAP1-GFP control (containing the empty pLZRS construct, ΔNGFR^+^); MJS TAP1-GFP UL49.5^BHV-1^ (containing BHV-1 UL49.5 SO, ΔNGFR^+^); MJS TAP1-GFP UL49.5^PRV^ (containing PRV UL49.5 SO, ΔNGFR^+^) and MJS TAP1-GFP UL49.5^EHV-1^ (containing EHV-1 UL49.5 SO, ΔNGFR^+^). To generate recombinant retroviruses for MDBK cell line transductions, the GP2-293 pantropic packaging cell line was used according to the protocol obtained from BD Bioscience Clontech (www.bdbiosciences.com). In brief, 1×10^6^ of GP2-293 cells were co-transfected with retroviral expression vector (pZLRS-IRES-GFP containing the BHV-1 UL49.5 gene in anti-sense or in the sense orientation) and pVSV-G construct (envelope vector) for retrovirus production. Retrovirus-containing medium was collected 48 hours post-transfection. MDBK cells were transduced four times with VSV-G containing recombinant retroviruses to generate the following stable cell lines: MDBK control (containing BHV-1 UL49.5 in anti-sense orientation, GFP^+^) and MDBK UL49.5^BHV-1^ (containing BHV-1 UL49.5 SO, GFP^+^). All cell lines generated in this study were selected for GFP or ΔNGFR expression using a FACSVantage cell sorter (Becton Dickinson). To obtain MJS cells stably expressing the HCMV-encoded US6 (MJS US6), MJS cells were transfected with pcDNA3-US6-IRES-NLS-GFP and selected for neomycin resistance [64].

### Reagents

The following antibodies were used in this study: anti-transferrin receptor (TfR) monoclonal antibody (mAb) 66Ig10, anti-TfR mAb H68.4 (Roche), anti-human MHC class I complexes mAb W6/32, anti-human MHC class I heavy chain mAb HC-10 (kindly provided by H. Ploegh, Whitehead Institute, Cambridge, Massachusetts, USA), anti-human class II HLA-DR mAb Tü36 (kindly provided by A. Ziegler, Institute for Immunogenetics, Universitätsklinikum Charité, Berlin, Germany), anti-TAP1 mAb 148.3 [54] and anti-TAP2 mAb 435.3 (kind gift from P. van Endert, Institut National de la Santé et de la Recherche Médicale, Paris, France). For the detection of equine TAP2, the polyclonal antibody anti-rat TAP2 Mac394 was used (kindly provided by M. Knittler, Institute of Immunology, Friedrich-Loeffler-Institute, Tübingen, Germany). For preparation of bovine TAP1 specific antibody, the bovine TAP1 ORF sequence encoding amino acid residues 117 to 167 were amplified from bovine genomic DNA and cloned into pGEX-4T-2 (GE Healthcare). The TAP1 polypeptide encompassing residues aa117-167 was purified as described previously [65]. The monoclonal antibody IL-A19 directed against bovine MHC class I molecules (a kind gift from Dr. J. Naessens, ILRAD, Nairobi, Kenya). The anti-equine and anti-canine MHC class I complexes mAb H58A and anti-porcine MHC class I mAb PT85A were purchased from VMRD Inc., Pullman, WA, U.S.A.

Mouse anti-BHV-1 UL49.5 serum was kindly provided by G.J. Letchworth (University of Wisconsin, Madison, Wisconsin, USA). Polyclonal rabbit anti-BHV-1 UL49.5 serum H11 was raised against a synthetic peptide representing the N-terminal sequence of BHV-1 UL49.5 and has been described [42]. In fig. 5, a different polyclonal rabbit anti-BHV-1 UL49.5 was used, obtained using a synthetic peptide corresponding to amino acid residues 27–41 of UL49.5 ([H] DAMRREGAMDFWSAGC*-[OH]). To facilitate conjugation to keyhole limpet hemocyanin, an additional irrelevant cysteine was added at the C terminus of the peptide (indicated by *). Rabbits were immunized by as described earlier [66]. The rabbit antiserum raised against PRV UL49.5 (gN) has been described [67], as was the anti-EHV-1 UL49.5 rabbit serum [43]. The VZV UL49.5-specific antibody was raised against two synthetic peptides: the N-terminal peptide EPNFAERNFWHASCSARGVYIDGSMITTLFKK and the C-terminal peptide RLFTRSVLRSTW. Both peptides were conjugated to glutathione S-transferase (GST) according to the methods described in [42]. The peptide-GST conjugates were mixed at a 1:1 ratio and emulsified in Freund's complete adjuvant for the first immunization and Freund's incomplete for the following immunizations. At 3-weeks intervals, the rabbit received four additional subcutaneous immunizations with the conjugates.

### Flow Cytometry

Cells were trypsinized and resuspended in phosphate-buffered saline (PBS) containing 1% bovine serum albumin (BSA) and 0.05% sodium azide. Cells were incubated with specific antibodies on ice for one hour. After washing, the cells were incubated with phycoerytrin (PE)-conjugated anti-mouse antibody for 45 min. Stained cells were analyzed by flow cytometry on a FACSCalibur flow cytometer (Becton Dickinson). To exclude dead cells, 7-aminoactinomycin D (7-AAD, Sigma-Aldrich) was added at a concentration of 0.5 µg/ml to all samples before analysis. Cells were analyzed using CellQuest software (Becton Dickinson).

### Peptide Transport Assay

The fluorescence-based peptide transport assay was performed as previously described [11],[68]. In brief, MJS cells were permeabilized with Streptolysin O (Murex Diagnostics Ltd.) at 37°C, followed by incubation with the fluorescein-conjugated synthetic peptide CVNKTERAY (N-core glycosylation site underlined) in the presence or absence of ATP. Peptide translocation was terminated by adding ice-cold lysis buffer containing 1% Triton X-100. After lysis, cell debris was removed by centrifugation, and supernatants were collected and incubated with Concanavalin A (ConA)-Sepharose (Amersham). After extensive washing of the beads, the peptides were eluted with elution buffer (500 mM mannopyranoside, 10 mM EDTA, 50 mM Tris-HCl pH 8.0) by vigorous shaking and further separated from ConA by centrifugation at 12,000×g for 2 minutes. The fluorescence intensity was measured using a fluorescence plate reader (CytoFluor, PerSeptive Biosystems; excitation 485 nm/emission 530 nm). The data were analyzed using the unpaired t-test. Statistical significance was set at p <0.05.

### Immunoprecipitations, Immunoblotting and Radiolabeling

Cells were lysed in a buffer containing 1% (wt/vol) digitonin, 50 mM Tris·HCl (pH 7.5), 5 mM MgCl_2_, 150 mM NaCl, 1mM leupeptin, and 1 mM AEBSF (4-(2-Aminoethyl)-benzenesulfonyl fluoride), and subjected to immune precipitations using anti-TAP1 mAb 148.3 o/n. To determine steady state protein levels, cells were lysed in Nonidet P-40 (NP-40) lysis mix containing 50 mM Tris-HCl, pH 7.4, 5 mM MgCl_2_ and 0.5% NP-40, supplemented with 1 mM AEBSF (4-(2-Aminoethyl)-benzenesulfonyl fluoride), 1 mM leupeptin and 20 µM Cbz-L3 (Carbobenzoxy-1-Leucyl-1-Leucyl-1-Leucinal-H; Peptides International, Inc). The samples were kept on ice throughout the experiment. Protein complexes were denatured in reducing sample buffer (2% SDS, 50 mM Tris pH 8.0, 10% glycerol, 5% β-ME, 0.05% bromophenol blue) for 5 min at 96°C. Immunoblotting (IB) analysis was performed on denatured cell lysates separated by SDS-PAGE and blotted onto polyvinylidene fluoride (PVDF) membranes. Blots were incubated with the antibodies as indicated, followed by horseradish peroxidase-conjugated goat-anti-mouse or swine-anti-rabbit Igs (DAKO and Jackson Laboratories), and visualized by ECLplus (Amersham).

Steady-state labeling of MDBK cells with [^35^S]-methionine/cysteine and subsequent immunoprecipitations with rabbit BHV-1 UL49.5-specific antibody were performed as described [69]. Immunoblotting procedures with rabbit anti-bovine TAP1 and rabbit anti-α-tubulin have been described [65].

### Cytotoxicity Assay

A total of 1,000 ^51^Cr-labeled target cells were incubated with different CD8^+^ CTL clones at various effector to target ratios. The HY-A1 clone recognizes an HY epitope in the context of HLA-A1, and the HA2.27 clone recognizes the histocompatibility antigen HA-2 in the context of HLA-A2. After 4 hours of incubation at 37°C, ^51^Cr release into the supernatant was measured using standard methods. The mean percentage of triplicate wells was calculated as follows: % specific lysis = (experimental release–spontaneous release)/(maximal release–spontaneous release)×100. For analysis of IFN-γ production, 20,000 T-cells were co-cultured with 10,000 target cells. After 24 hours, the supernatant was harvested and the concentration of IFN-γ was measured by standard ELISA (Sanquin, Amsterdam, The Netherlands).

### Viruses and Virus Infections

The wild type viruses used in this study were: BHV-1 strain Lam, BHV-1 strain Cooper (Fig. 5), PRV strain Kaplan and EHV-1 strain RacL1.The UL49.5 deletion mutant of PRV used in this study has been described before [41],[43]. The UL49.5 deletion mutant of EHV-1 (strain RacL11) was a gift from J. von Einem (College of Veterinary Medicine, Cornell University, Ithaca, NY, USA). Infections with wild type and mutant herpesviruses were carried out on the following cell lines: MDBK cells for BHV-1; PK15 cells for PRV and E.derm cells for EHV-1. The cells were washed once with PBS and infected with BHV-1 or PRV at an m.o.i. of 10, and with EHV-1 at an m.o.i. of 5 at 37°C in serum-free medium. After 2 hours, medium containing 10% FBS was added. Mock-infected cells were treated under the same conditions as infected cells. After 5 hours of infection, cells were collected and prepared for the peptide translocation assay. For immunoblotting and metabolic labeling experiments, MDBK cells were infected with BHV-1 wt and UL49.5Δtail viruses for 12 hours.

### Construction of BHV-1 UL49.5 recombinant viruses

The BHV-1 UL49.5 mutant was generated by homologous recombination, using BHV-1 strain Lam as parent strain. The recombination region upstream of the UL49.5 gene was a 1.4 kb fragment running from nucleotide residue 7670 to 9061 (residue numbers based on the complete BHV-1 genome with NCBI accession number NC_001847, updated 30 March 2006). This fragment starts at a *Bst*XI site 1.3 kb upstream of the start codon of the UL49.5 open reading frame and ends at its amino acid residue 31. The recombination region downstream of the UL49.5 gene was provided by a 1.9 kb fragment from nucleotide residue 9075 to 10972. This fragment starts at amino acid residue 36 of UL49.5 and ends at an *Fsp*I site 1.7 kb downstream of the UL49.5 open reading frame. A 2.2 kb *Nru*I–*Pvu*II fragment was cloned between the two UL49.5 recombination fragments that carries the hGFP gene in the expression cassette of pcDNA3 (Invitrogen). The complete recombination fragment (5.5 kb) was co-transfected with purified BHV-1 Lam DNA into EBTr cells using a calcium phosphate-based transfection method. After plating the supernatant of freeze/thawed transfected cells, a green plaque was found that, following three rounds of plaque purification, failed to react with anti-BHV-1 UL49.5 serum. The BHV-1-UL49.5 mutant could be grown to a titer of 10^7.0^ TCID_50_/ml and was capable of penetrating bovine cells with the same kinetics as the wild type Lam strain.

The BHV-1 recombinant virus gN Am80, expressing a form of UL49.5 lacking its cytoplasmic domain, was constructed by introducing an amber mutation at gN residue 80R (AGG to TAG) by using a BHV-1 BAC clone (Liu and Chowdhury, manuscript in preparation).

### Peptide Binding Assay

Cellular microsomes were prepared as described [70]. Microsomes isolated from 7×10^6^ homogenized cells were pre-incubated in 50 µl of AP buffer (5 mM MgCl_2_ in phosphate-buffered saline, pH 7.0) on ice for 45 min in the absence or presence of a 200-fold molar excess of the non-labeled TAP-specific viral inhibitor ICP47 [18]. Different concentrations of radiolabeled peptide (RR[^125^I]YQKSTEL) were added equally to the samples with or without ICP47 and incubated on ice [71]. Non-bound peptides were removed by washing the membranes with 400 µl of AP buffer and subsequent centrifugation at 20,000 g for 8 min. The amount of radioactivity bound to the membranes was quantified by γ-counting and corrected for the signal obtained in the presence of ICP47. All experiments were performed in triplicate.

### ATP-Agarose Binding Assay

TAP binding to ATP-agarose was assayed as described [11]. In brief, cells were solubilized in 1% (w/v) digitonin, 50 mM Tris-HCl (pH 7.5), 5 mM MgCl_2_, 150 mM NaCl, 5 mM iodoacatamide, and 1 mM AEBSF. Hydrated C-8 ATP-agarose (Fluka/Sigma) was added to the post-nuclear supernatant and incubated by rotation at 4°C. After 2 hours, the supernatant was separated from the ATP-agarose pellet by 5 minutes centrifugation. The resulting pellet was washed three times with 0.1% (w/v) digitonin, 50 mM Tris-HCl (pH 7.5), 5 mM MgCl_2_ and 150 mM NaCl. Proteins bound to the ATP-agarose were eluted with 500 mM EDTA and SDS sample buffer was added to both the supernatant and the pellet. The samples were separated using SDS-PAGE and analyzed by immunoblotting.

### FRAP

Confocal microscopy and Fluorescence Recovery After Photobleaching (FRAP) assays were performed as described [11],[57]. In short, a circular spot in the ER was bleached at full intensity, and an attenuated laser beam was used to monitor recovery of fluorescence. The half-time for recovery was calculated from each recovery curve after correction for loss of fluorescence caused by imaging (usually <4%). The diffusion coefficient D was determined from at least seven cells measured in different experiments.

### Accession numbers

UL49.5 sequence data used to generate the alignment shown in Fig. 1 and the phylogenetic tree shown in Fig. 7, have been obtained from the NCBI (www.ncbi.nlm.nih.gov) database with the accession numbers: bovine herpesvirus 1 (BoHV-1) [NP_045309], bovine herpesvirus 5 (BoHV-5) [NP_954898], cercopithecine herpesvirus 1 (CeHV-1) [AAP41468], cercopithecine herpesvirus 9 (CeHV-9) [NP_077423], cercopithecine herpesvirus 16 (CeHV-16) [YP_443897], equid herpesvirus 1 (EHV-1) [AAT67267], equid herpesvirus 4 (EHV-4) [CAA35670], gallid herpesvirus 1 (GaHV-1) [YP_182341], gallid herpesvirus 2 (GaHV-2) [NP_057812], gallid herpesvirus 3 (GaHV-3) [NP_066882], human herpesvirus 1 (HSV-1) [NP_044652], human herpesvirus 2 (HSV-2) [NP_044520], human herpesvirus 3 (VZV) [YP_068406], meleagrid herpesvirus 1 (MeHV-1) [AAG30090], psittacid herpesvirus 1 (PsHV-1) [AAQ73691], suid herpesvirus 1 (PRV) [YP_068325], transporter 1 ATP-binding cassette sub-family B [*Bos taurus*] [AAY34698]. Not obtained from the NCBI database are: bubaline herpesvirus 1 (BuHV-1)[MSRSLLVALATAALLAMVRGLDPLLDAMRREE AMDFWSAGCYARGVPLSEPPQAMVVFYAALTVVMLAVALYAYGLCFRLMSAGGPNKKEVRGRG; FAMR, unpublished], canid herpesvirus (CHV) [patent EPO910406 http://ep.espacenet.com ], cervid herpesvirus 1 (CvHV-1) [MARMPRSLLSALAVAALLAIAGARDPLLDAMRHEGAMDFWSASCYARGVPL SEPPQALVVFYVALAVVMFSVAVYAYGLCLRLVGADSPNKKDSRGRG; FAMR, unpublished].
